# Supplementary material for: Inheritance of Resistance to Cry1A.105 in Helicoverpa zea (Boddie) (Lepidoptera: Noctuidae)
Source: Insects. 2022 Sep 27;13(10):875. doi: 10.3390/insects13100875 (PMC9604160; doi:10.3390/insects13100875)
Supplement: Supplementary file 1 [file insects-13-00875-s001.zip › insects-1900739-supplementary.pdf]

Supporting Information (SI)

Table S1. Larval mortality (% mean ± sem) of different genetic populations of *H. zea* at four Cry1A.105 concentrations in diet-overlay bioassays*.

**Insect Population**	**Cry1A.105 Concentration (μg/cm²)**			
	**1.00**	**3.16**	**10.00**	**31.60**
		Test-I		
BZ-SS	100 ± 0.0 a	100 ± 0.0 a	100 ± 0.0 a	Not available
Cry1A.105-RR	0.0 ± 0.0 g	17.2 ± 8.7 f	31.3 ± 6.8 ef	60.9 ± 3.0 bcd
Cry1A.105-F_{1a}	33.3 ± 9.2 def	50.8 ± 9.1 cde	79.4 ± 6.0 bc	89.8 ± 1.5 ab
Cry1A.105-F_{1b}	55.2 ± 8.3 cde	65.9 ± 4.0 bcd	68.0 ± 7.8 bc	79.2 ± 4.8 bc
Pooled F_1	44.3 ± 7.1	58.3 ± 5.5	73.7 ± 5.0	84.5 ± 3.5
ANOVA	$F_{3,45}$ = 159.76, P < 0.0001 for population; $F_{3,45}$ = 35.91, P < 0.0001 for Bt concentration; and $F_{8,45}$ = 4.64, P = 0.0004 for interaction.			
		Test-II		
BZ-SS	100 ± 0.0 a	100 ± 0.0 a	100 ± 0.0 a	Not available
Cry1A.105-RR′	21.0 ± 2.5 h	28.6 ± 4.6 gh	48.1 ± 2.5 efg	52.8±6.1 efg
Cry1A.105-$F_{1'a}$	28.3 ± 4.5 gh	28.9 ± 2.7 gh	51.6 ± 4.1 efg	67.2 ± 6.9 b-e
Cry1A.105-$F_{1'b}$	20.4 ± 6.5 h	35.9 ± 3.0 fgh	60.2 ± 5.5 b-f	68.8 ± 6.8 b-e
Pooled $F_{1'}$	24.3 ± 4.0	32.4 ± 2.3	55.9 ± 2.6	68.0 ± 4.5
Cry1A.105-$F_{2'}$	35.2 ± 3.2 fgh	53.6 ± 6.5d-g	60.0 ± 3.5 c-f	84.4 ± 3.1bc
Cry1A.105-BC′	62.5 ± 7.1 b-f	64.1 ± 7.0 b-f	78.9 ± 5.5 bcd	85.9 ± 3.9 b
ANOVA	$F_{5,85}$ = 139.88, P < 0.0001 for insect population; $F_{3,85}$ = 54.04, P < 0.0001 for Bt concentration; and $F_{14,85}$ = 1.84, P = 0.0459 for interaction			

* Means followed by the same letter within each test are not significantly different (LSMEANS tests, α = 0.05). If a mean was followed by four or more letters, an abbreviation with only the first and last letters are presented; for example, ’b-f’ means ‘bcdef’.
